# Batch and semi-continuous microalgal TAG production in lab-scale and outdoor photobioreactors

**DOI:** 10.1007/s10811-016-0897-1

**Published:** 2016-07-07

**Authors:** Giulia Benvenuti, Rouke Bosma, Fang Ji, Packo Lamers, Maria J. Barbosa, René H. Wijffels

**Affiliations:** 1 Bioprocess Engineering, AlgaePARC, Wageningen University, P.O. Box 16, 6700 AA Wageningen, The Netherlands; 2 Biomass Engineering Center, China Agricultural University, P.O. Box 50, Beijing, 100083 China; 3 Biosciences and Aquaculture, Nordland University, N-8049 Bodø, Norway

**Keywords:** Microalgae, TAG production, Batch, Semi-continuous, Outdoor

## Abstract

Microalgal triglycerides (TAGs) represent a sustainable feedstock for food, chemical and biofuel industries. The operational strategy (batch, semi-continuous, continuous cultivations) has an impact on the TAG productivity. In this study, semi-continuous (i.e. with fixed harvesting frequency) and batch cultivations were compared on TAG production both at lab-scale and in outdoor cultivations. At lab-scale, the semi-continuous TAG productivity was highest for a cycle time of 2 days (SC1; 0.21 g L^−1^ day^−1^) and similar to the maximum obtained with the batch (optimal harvest time; 0.23 g L^−1^ day^−1^). Although TAG content was lower for SC1 (22 %) than for the batch (35 %), higher biomass productivities were obtained with SC1. Outdoors, semi-continuous cultivations were subjected to a lower degree of stress (i.e. higher amount of nitrogen present in the system relative to the given irradiance) compared to lab-scale. This yielded low and similar TAG contents (10–13 %) in the different semi-continuous runs that were outdone by the batch on both TAG content (15–25 %) and productivity (batch, 0.97–2.46 g m^−2^ day^−1^; semi-continuous, 0.35–0.85 g m^−2^ day^−1^). The lab-scale experiments showed that semi-continuous strategies, besides leading to similar TAG productivities compared to the batch, could make TAG production cost effective by valorising also non-TAG compounds. However, optimization of outdoor semi-continuous cultivations is still required. For instance, the nitrogen supply and the harvest frequency should be adjusted on the total irradiance. Additionally, future research should focus on recovery metabolism upon nitrogen resupply.

## Introduction

Under adverse growth conditions, microalgae can accumulate high amounts of fatty acids in the form of triglycerides (TAGs). Microalgal TAGs are increasingly discussed as sustainable feedstock for the commodity markets (i.e. food, chemical and biofuel) (Wijffels et al. 2010; Mata et al. 2010; Draaisma et al. 2013). Microalgae as TAG cell factories offer several advantages over agricultural crops, which are currently used to produce those commodities. Besides producing valuable co-products (Mulders et al. 2014), microalgae can be cultivated on non-arable land and they have a low freshwater and fertilizer footprint when grown on wastewaters, sea- or brackish water. Most importantly, higher TAG productivities can be obtained with microalgae compared to agricultural crops (Chisti 2007; Hu et al. 2008).

At lab-scale, under defined conditions (e.g. temperature, light), high TAG productivities have already been achieved with some microalgal species (Griffiths and Harrison 2009; Breuer et al. 2012; Ho et al. 2014a; Benvenuti et al. 2014). However, it should always be validated whether the productivities obtained at lab-scale can be translated to outdoor cultivations, in which cells are subjected to varying (e.g. light, temperature) conditions. For this reason, outdoor pilot-scale research is essential to identify technical and process bottlenecks that should be tackled before scaling up.

When producing microalgal TAGs, an important aspect to evaluate is the adopted operational strategy (i.e. batch, semi-continuous, continuous cultivations), because it strongly affects process productivity (Benvenuti et al. 2015). Presently, TAG production is widely carried out in a two-step batch process (Zemke et al. 2010; Feng et al. 2011; Münkel et al. 2013; San Pedro et al. 2014) in which biomass is firstly produced under nitrogen replete conditions, and subsequently subjected to nitrogen (N) starvation to trigger TAG accumulation. A batch process, besides being easy to operate, ensures high TAG contents (>30 % *w*/*w*). However, after reaching a maximum within the first days of cultivation, TAG productivity decreases, due to a declining photosynthetic activity during N-starvation (Breuer et al. 2012; Benvenuti et al. 2014). Additionally, at the start of the batch, a fraction of the facility area and time are invested in inoculum production rather than in actual TAG production. Finally, a batch process implies downtime for reactor cleaning and startup in between runs, thus decreasing productivity and increasing labour, water and chemical demands.

These disadvantages possibly can be overcome by semi-continuous (Rodolfi et al. 2009; Bondioli et al. 2012) and continuous (Klok et al. 2013; Lucas-Salas et al. 2013; Wen et al. 2014) cultivations. Despite these operational modes are more complex to operate, they offer several advantages (Klok et al. 2014). Firstly, maximum TAG productivities, obtained within the first hours/days of batch cultivations, can potentially be maintained for longer periods in optimized (semi)- continuous processes. Secondly, cultivation settings (e.g. cycle duration) can be adjusted to changing light conditions. Finally, biomass production and TAG accumulation occur simultaneously in the same reactor, and downtime is negligible for long-term runs. Therefore, semi-continuous or continuous processes could result in a stable and robust process with higher TAG productivity compared to the classical batch approach.

Recent advances for (semi)-continuous TAG production have been reported (Bona et al. 2014; Terigar and Theegala 2014; Wen et al. 2014; Ho et al. 2014b). However, to develop a robust alternative process, it is necessary to perform a solid comparison of (semi)-continuous and batch strategies under exactly the same cultivation conditions (e.g. reactor design, light regime). Additionally, it is very important to perform process comparison not only under defined lab-scale conditions but also outdoors under changing weather conditions, and assess whether the findings obtained at lab-scale can be translated to outdoor cultivations.

The aim of this study was to investigate semi-continuous processes both at lab-scale and in outdoor cultivations and compare them to batch processes on TAG productivity.

## Materials and methods

### Growth medium

In all pre- and cultivation steps both at lab-scale and outdoors, cells were grown on a medium constituted of disinfected and filtered natural seawater (Oosterschelde, the Netherlands; (Benvenuti et al. 2015)) enriched with a nutrient stock consisting of (in mM): HEPES (for pre-cultivation in Erlenmeyer flasks), 20; KH_2_PO4, 1.7; Na_2_EDTA, 0.56; FeSO_4_·7H_2_O, 0.11; MnCl_2_·2H_2_O, 0.01; ZnSO_4_·7H_2_O, 2.3 × 10^−3^; Co(NO_3_)_2_·6H_2_O, 0.24 × 10^−3^; CuSO_4_·5H_2_O, 0.1 × 10^−3^; Na_2_MoO_4_·2H_2_O, 1.1 × 10^−3^; NaNO_3_, 25 (for pre-cultivation in Erlenmeyer flasks). During cultivation in reactors, nitrogen was supplied as described in “Experimental approach” section.

### Experimental approach

Batch and semi-continuous cultivations were tested both under defined lab-scale conditions and in outdoor reactors and compared on TAG productivity.

**Fig. 1:**
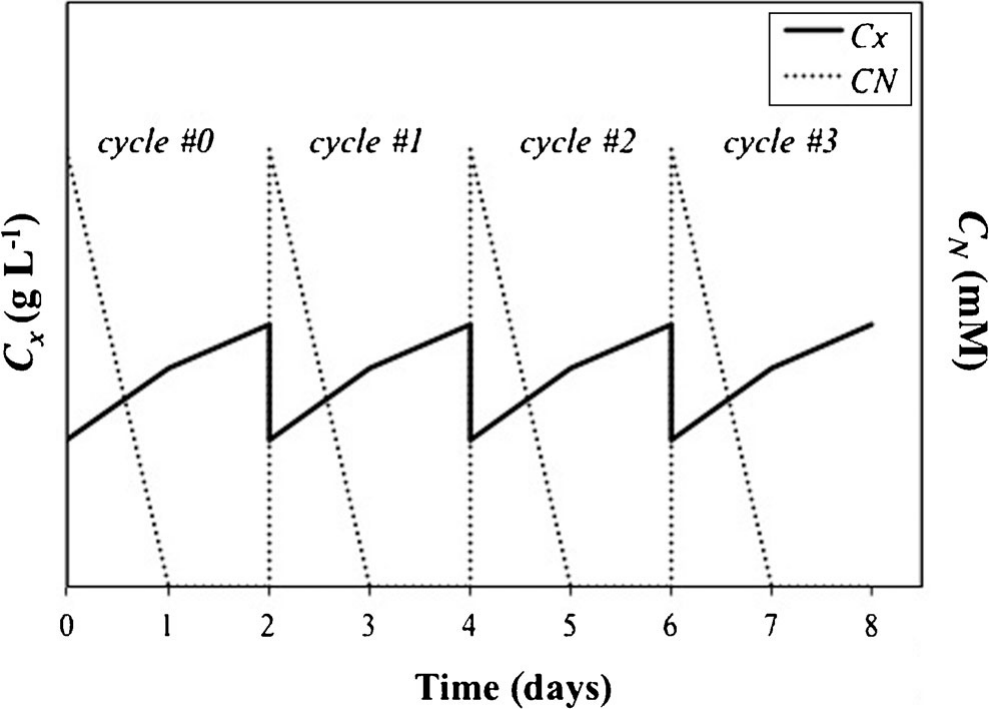
Schematic representation of a semi-continuous cultivation

Medium containing nitrogen (N) was added at the start of the batch cultivations. At N-depletion, the cultures were supplied with a N-free stock to prevent limitation of nutrients other than nitrogen, and subsequently cultured for 10 days. In the adopted semi-continuous strategy (Fig. 1), cells were inoculated in N-replete conditions (day 0 in Fig. 1). At N-depletion (day 1 in Fig. 1), TAG accumulation commenced. After a fixed number of days from N-depletion (1, 2 or 3 days), the culture was partly harvested (day 2 in Fig. 1) and replenished with medium. In both lab-scale and outdoor cultivations, the harvested volume was chosen as such that the next cycle started at 1 g L^−1^. Nitrogen was dosed in the medium as such that each cycle always started with 5 mM (lab-scale) and 2.5 mM (outdoors) of extracellular N. This ensured a re-growth phase that continued until extracellular N was depleted again. At that point, a new TAG-accumulation phase followed until a new harvest was applied.

To harvest the culture at the right frequency (i.e. 1, 2 or 3 days after the onset of N-depletion), preliminary tests were conducted to identify the time at which external N-NO_3_^−^ concentration was zero. Typically, this was at 24 h after addition of nitrogen for both lab-scale and outdoor runs.

#### Lab-scale cultivations

##### Inoculum production and cultivation conditions

Pre-cultures of *Nannochloropsis* sp. CCAP 211/78 were maintained in 250 mL Erlenmeyer flasks, which were placed in an orbital shaker incubator (Multitron, Infors HT, The Netherlands) at 120 rpm under 2 % CO_2_-enriched headspace, 70 % humidity. The flasks were continuously illuminated at a light intensity of 50 μmol photons m^−2^ s^−1^ supplied by fluorescent lamps (TL-D Reflex 36 W/840, Philips, The Netherlands). Two-week-old flask cultures were centrifuged (780×*g*, 5 min) to remove remaining nutrients. Subsequently, cells were re-suspended in medium and inoculated in an airlift-loop photobioreactor with a light path of 20.7 mm, 1.9 L working volume and 0.08 m^2^ surface area (Labfors, Infors HT, 2010). Mass-flow controllers supplied 1.0 L min^−1^ pressurized air for mixing. The pH was set at 7.5 and controlled by means of on-demand CO_2_ addition. A culture temperature of 25 °C was maintained by water recirculation through water jackets that were in direct contact with the reactor cultivation chamber.

Each semi-continuous was stopped when three consecutive cycle repetitions were achieved (i.e. constant biomass concentration and TAG content at harvest), whereas the batch culture was kept for 10 days after the onset of N-depletion.

##### Light supply

For the first cultivation days, the ingoing light intensity was increased daily to keep the outgoing light at about 20 μmol photons m^−2^ s^−1^. When the biomass concentration reached 0.7–0.9 g L^−1^, simulated day/night light rhythms of a midsummer day in the Netherlands were applied. By applying sinusoidal functions (Eq. 1), sunrise and sunset were simulated between 6 AM and 10 PM. The light intensity gradually increased and reached the maximal value (1500 μmol photons m^−2^ s^−1^) at 2 PM, after which it decreased to zero again.

1 $$ E(t)= \sin \left(\frac{t}{P}\cdotp\ \pi \right)\cdotp {E}_{\max } $$

In which *t* is the number of hours after sunrise (hours); *E*_max_ is the maximum light intensity (μmol photons m^−2^ s^−1^), *P* is the duration of the light period (hours).

#### Outdoor cultivations

Semi-continuous TAG production processes were also tested under outdoor conditions and their ground areal TAG productivities were compared to those achieved with batch cultivations. The two operational strategies were tested at AlgaePARC pilot facilities in Wageningen, the Netherlands (N 51°59′45 88″, 5°39′28.15″) over different seasons (July–October 2014) in identical vertically stacked horizontal tubular reactors (VRs; 170 L culture volume, 4.4 m^2^ ground area) which were simultaneously operated.

##### Inoculum production and cultivation conditions

Pre-cultures were maintained in 250 mL Erlenmeyer flasks, as previously described. The flask cultures were used to inoculate a 20-L panel reactor with a 4-cm light path. Mass-flow controllers (Brooks Instrument LLC 0254, Hungary) supplied 1.50 L min^−1^ pressurized air for mixing, as well as CO_2_, which ensured a culture pH of 7.5. A temperature of 25 °C was maintained by water recirculation through heating coils. An ingoing irradiance of 350 μmol photons m^−2^ s^−1^ was supplied by fluorescence tubes placed in front of the reactor. From this flat panel reactor, a 1-week-old culture was used to inoculate an outdoor horizontal tubular reactor (90 L) (Benvenuti et al. 2015) operated as turbidostat at 3 g L^−1^. The biomass produced in this horizontal tubular reactor was used to inoculate the two identical VRs at similar starting biomass concentration (0.5–0.8 g L^−1^) in N-free medium. One system was operated as batch and the other system as semi-continuous.

##### Operational settings for the outdoor reactors

In the three outdoor tubular reactors (HR, VR1 and VR2), liquid velocity was set at 0.34 m s^−1^. To keep the pH at 7.5, CO_2_ was added to the culture on demand. Temperature was kept between 20 and 30 °C by means of valves (Proportional Integral Differential regulation) that allowed either warm water (max. 60 °C) or chilled water (8 °C) to flow through a double-walled stripper, heating up or cooling down the culture until the set point was reached. A detailed description of the outdoor systems is given by Bosma et al. (2014) and Benvenuti et al. (2015).

In both VRs, the residual nitrogen (N) carried along with the inoculum supported about 0.5 g L^−1^ of newly formed biomass. In such a way, an initial biomass concentration for the TAG-accumulation phase of 1.0–1.5 g L^−1^ was reached. These initial biomass concentrations were chosen based on the findings of our previous study (Benvenuti et al. 2015) which identified it as the most suitable range to achieve high TAG productivities with *Nannochloropsis* sp. cultivated during summer and fall in outdoor vertically stacked tubular reactors in the Netherlands. Nitrogen was depleted from the medium within the first 2–4 days of cultivation in VRs, depending on the light received in this initial period. The moment of N-depletion was considered as start of the N-starvation phase for the batch and of cycle #0 for the semi-continuous cultivation.

The semi-continuous cultures were harvested and diluted to the set turbidity value (i.e. biomass concentration, 1 g L^−1^) by means of harvest and medium supply pumps (Bosma et al. 2014). Offline dry weight determinations were used to calibrate the response curve of turbidity. In all systems, a linear relation of dry weight concentrations and turbidity was found with high accuracy (*R* ^2^ > 0.90).

For the semi-continuous cultivations, we aimed to harvest the culture at maximum ground areal TAG productivity. Because it was expected that more time is required when less light is available (Benvenuti et al. 2015), lower harvest frequencies were chosen when lower total irradiance was expected (Table 1).

**Table 1:** Operational period, corresponding time-averaged light intensity (*E*_ground_ (*t*)), days from N-depletion at which harvest was applied and number of harvest events are reported. *SC*, semi-continuous; *B*, batch

Run	Operational period (2014)	*E*_ground_ (*t*) (mol photons m^−2^ day^−1^)	# days harvest from N-depletion	# harvest events
SC1	16 July–8 August	37	1	9
B1*a*	12–24 July	39	10	1
B1*b*	26 July–6 August	36	9	1
SC2	19–28 August	26	2	3
B2	15–27 August	23	10	1
SC3	10 September–6 October	20	3	5
B3*a*	3–17 September	24	10	1
B3*b*	20 September–4 October	18	10	1

The batch cultivation was kept for 10 days after N-depletion, after which the complete reactor was harvested and cleaned before repeating the process again. For the semi-continuous cultivation, the same harvest frequency was tested for about a month (Table 1). Exception was the semi-continuous run carried out in the second half of August (SC2). This run was stopped after 14 days because the biofilm formed in the tubes heavily impaired light penetration through the culture.

### Offline measurements

Biomass samples were taken between 9 AM and 10 AM from the outdoor cultivations and at 2:00 PM from the lab-scale ones. Biomass concentration was measured daily (optical density 750 nm and dry weight), whereas cellular TAG content was measured only in harvested biomass. Dry weight was determined as described by Vejrazka et al. (2011) and cellular TAG content was analysed as described by Breuer et al. (2012) and Breuer et al. (2013a). Residual N-NO_3_^−^ in the medium was measured daily, until its depletion, with an AQ2 nutrient analyser (Seal Analytical, USA) as described by Benvenuti et al. (2015).

### Calculations and definitions

#### Time-averaged biomass and TAG productivity for batch and semi-continuous cultivations

Time-averaged volumetric biomass and TAG productivity (*P*_*j,* vol_ *(t)*; g L^−1^ day^−1^) was calculated according to Eq. 2;

2 $$ {P}_{j,\ \mathrm{vol}}(t) = \frac{{\displaystyle {\sum}_{t=0}^t}\ \left({H}_j\right)\ }{V_{\mathrm{R}}\ \cdotp\ {t}_i\ } $$

In which *H*_*j*_ (g) is the amount of biomass or TAGs present in the harvest (for batch cultivations, *H*_*j*_ was calculated with the total reactor harvest); *V*_R_ is the reactor volume (L); *t*_*i*_ is any time point during cultivation (days).

To calculate TAG productivity of the batch cultivations, besides the N-starvation period (*t*_*i*, N-starvation_) also downtime (i.e. reactor cleaning and startup; *t*_downtime_) and inoculum production (i.e. amount of biomass present at the moment of N-depletion; *t*_*i*,__inoculum_) were considered. The following assumptions were made: downtime was fixed to 1 day, and inoculum was produced at a certain biomass yield per mole photons in a hypothetical “growth” reactor operated in continuous mode in nitrogen replete conditions. This hypothetical growth reactor supplied biomass to the batch reactor, which was subsequently subjected to N-starvation to trigger TAG accumulation. Hence, *t*_*i*, inoculum_ was calculated using the average light supplied rate over the cultivation period (Table 2) and an average biomass yield per mole photons of 0.59 g mol^−1^ photons. This yield was found for the most efficient outdoor biomass production system (i.e. flat panel PBR operated with a daily dilution rate of 0.27 day^−1^ over a period of 36 days) at AlgaePARC pilot facility, the Netherlands (de Vree et al. 2015). Thus, the time considered for calculations of batch time-averaged productivities is defined as *t*_*i*, batch_ = *t*_downtime_ + *t*_*i*,__inoculum_ + *t*_*i*, N-starvation_.

**Table 2:** Time for inoculum production (*t*_inoculum_), average light supply rate, reactor area (*A*_R_) and inoculum concentration (*C*_*x*, inoculum_) for the lab-scale and outdoor batch runs

	B*I*, _lab-scale_	B*II*, _lab-scale_	B1*a*_, out_	B1*b*_, out_	B2_, out_	B3*a*_, out_	B3*b*_, out_
*t*_inoculum_ (days)	1.4	1.3	1.9	2.0	3.1	2.7	3.3
Average light supply rate (mol photons day^−1^)	4.4	4.4	172	160	102	105	78
*A*_R_^a^ (m^2^)	0.08	0.08	4.4	4.4	4.4	4.4	4.4
*C*_*x*, inoculum_ (g L^−1^)	1.92	1.84	1.12	1.10	1.10	0.99	0.91

^a^ *A*_R_ is the illuminated reactor surface area for the lab-scale systems and the reactor ground area for the outdoor systems

For semi-continuous cultivations, both the startup procedure and the inoculum production will take place only at the beginning of the process and this time is negligible for long-term runs. Additionally, for the semi-continuous productivity, the first harvest (cycle #0) was not taken into account. As it was produced from N-replete biomass, it was not representative for a long-term operation. Thus, the start of cycle #1 was considered as start of the semi-continuous cultivations.

#### Time-averaged ground areal biomass and TAG productivity

For the outdoor runs, time-averaged ground areal biomass or TAG productivity (*P*_*j*_, _ground_ (*t*); g m^−2^ day^−1^) was calculated multiplying the time-averaged volumetric productivities by the reactor volume (170 L)-to-ground area (4.4 m^2^) ratio.

#### Biomass and TAG productivity over a semi-continuous cycle

For the semi-continuous cultivations, biomass and TAG productivity over a cycle (*P*_*j*, cycle_; g m^−2^ day^−1^) is also discussed. *P*_*j*, cycle_ was calculated by dividing the harvested biomass or TAGs (*H*_*j*_; g) at the end of the cycle by the reactor ground area (m^2^) and cycle duration (days).

#### Time-averaged biomass and TAG yields per mole photons

Time-averaged biomass (*Y*_*x*, ph_ (*t*); g mol^−1^ photons) and TAG (*Y*_TAG, ph_ (*t*); g mol^−1^ photons) yield per mole photons were calculated by dividing the time-averaged ground areal biomass or TAG productivity by the time-averaged irradiance (*E*_ground_ (*t*); mol photons m^−2^ day^−1^) received on ground area during the considered time interval.

## Results

### Lab-scale cultivations

#### Lab-scale batch cultivations

**Fig. 2:**
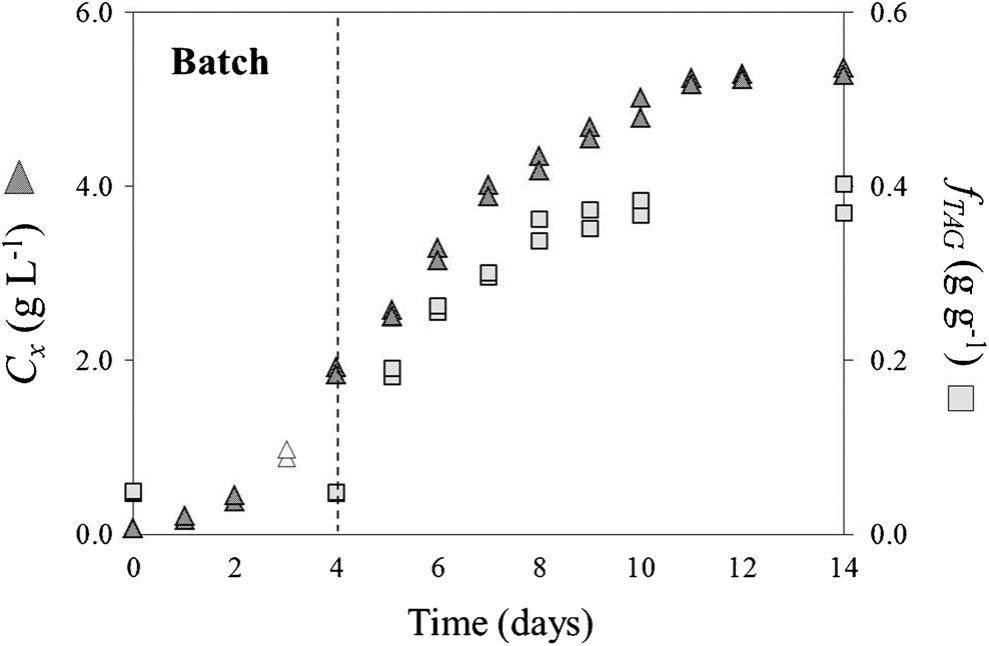
Time-evolution of biomass concentration and TAG content for the duplicate lab-scale batch cultivations. *Empty symbols* represent the day at which light intensity was switched to set point. The *dotted line* indicates day zero of nitrogen-starvation

Nitrogen (N) depletion (day 4) triggered accumulation of TAGs, which, within 24 hours, increased fourfold (Fig. 2). TAG content steadily increased until stabilizing at about 0.39 g g^−1^ by the end of the cultivation. Maximum time-averaged volumetric TAG productivity (*P*_TAG, vol, max_ (*t*); 0.23 g L^−1^ day^−1^) and yield per mole photons (*Y*_TAG, ph, max_ (*t*); 0.10 g mol^−1^ photons) were observed after 4 days of N-depletion (day 8) (Table 3). At *P*_TAG, vol, max_ (*t*), cellular TAG content was 0.35 g g^−1^.

**Table 3:** Time-averaged volumetric biomass productivity (*P*_*x*, vol, cycle_ (*t*)) and TAG (*P*_TAG, vol, cycle_ (*t*)) productivities, time-averaged biomass (*Y*_*x*, ph, cycle_ (*t*)) and TAG (*Y*_TAG, ph, cycle_ (*t*)) yields per mole photons over cycle and TAG content (*f*_TAG_) for the lab-scale batch and semi-continuous runs. Values for the each batch duplicate culture are shown. For the semi-continuous runs, mean and standard deviation are reported (*n* = 3). SC1, SC2 and SC3 were harvested every 1, 2 and 3 days after nitrogen-depletion, respectively

Lab-scale runs				
	Batch^a^	SC1	SC2	SC3
*P*_*x*, vol_ (*t*) (g L^−1^ day^−1^)	0.68 0.66	0.94 ± 0.02	0.57 ± 0.02	0.43 ± 0.01
*Y*_*x*, ph_ (*t*) (g mol^−1^ photons)	0.29 0.28	0.40 ± 0.01	0.25 ± 0.01	0.19 ± 0.00
*f*_TAG_ (g g^−1^)	0.34 0.36	0.22 ± 0.00	0.28 ± 0.01	0.33 ± 0.01
*P*_TAG, vol_ (*t*) (g L^−1^ day^−1^)	0.23 0.24	0.21 ± 0.01	0.16 ± 0.00	0.14 ± 0.00
*Y*_TAG, ph_ (*t*) (g mol^−1^ photons)	0.10 0.10	0.09 ± 0.00	0.07 ± 0.00	0.06 ± 0.00

^a^At maximum time-averaged TAG productivity

#### Lab-scale semi-continuous cultivations

**Fig. 3:**
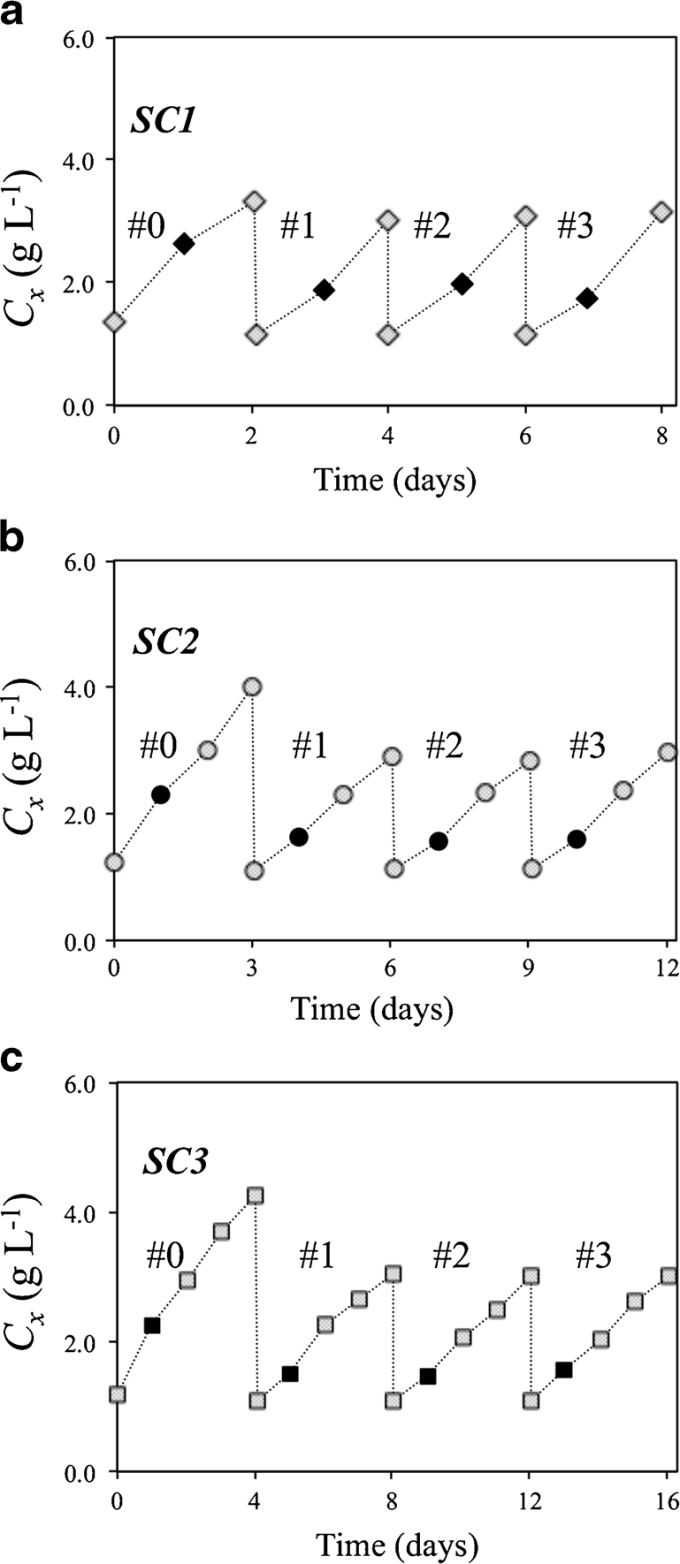
Time-evolution of biomass concentration for the lab-scale semi-continuous cultivations (SC1, SC2 and SC3). SC1, SC2 and SC3 were harvested every 1, 2 and 3 days after nitrogen-depletion, respectively. *Black symbols* indicate biomass concentration at N-depletion. Lines are drawn only for illustrative purposes

Each semi-continuous run was stopped after three consecutive and constant cycle repetitions (Fig. 3 and Table 3) (i.e. steady-state cycles; #1–#3). At the harvest of the constant cycle repetitions, biomass concentrations and TAG contents were equal for the different cycles (standard deviation within 5 % of average).

TAG productivity (0.14–0.21 g mol^−1^ photons) increased with decreasing cycle duration, whereas an opposite trend was observed for TAG content, which ranged from 0.22 to 0.33 g g^−1^ (Table 3).

### Outdoor cultivations

#### Outdoor batch cultivations

For the five outdoor batch cultivations, N-depletion occurred at 1.04 ± 0.09 g L^−1^, after which TAG accumulation commenced.

The highest maximum time-averaged TAG yield per mole photons (*Y*_TAG, ph, max_ (*t*); i.e. optimal harvest time for the batch), corresponding to 0.09 g mol^−1^ photons, was observed for the runs B2 and B3*a* (Table 4), which were performed at intermediate irradiance (23–24 mol photons m^−2^ day^−1^). At *Y*_TAG, ph, max_ (*t*), TAG content was 0.15–0.21 g g^−1^.

**Table 4:** Time-averaged ground areal biomass (*P*_*x*, ground_ (*t*)) and TAG (*P*_TAG, ground_ (*t*)) productivities, time-averaged biomass (*Y*_*x*, ph_ (*t*)) and TAG (*Y*_TAG, ph, max_ (*t*)) yields per mole photons, TAG content (*f*_TAG_) and time-averaged light intensity (*E*_ground_ (*t*)) for the outdoor batch runs (B1*a*, B1*b*, B2, B3*a*, B3*b*). In parentheses, the day of nitrogen-starvation at which maximum time-averaged TAG yield per mole photons was found

Outdoor batch runs					
	B1*a*	B1*b*	B2	B3*a*	B3*b*
*P*_*x*, ground_ (*t*)^a^ (g m^−2^ day^−1^)	11.85	9.23	8.99	11.45	6.33
*Y*_*x*, ph_ *(t*)^a^ (g mol^−1^ photons)	0.36	0.28	0.39	0.46	0.40
*f*_TAG_^a^ (g g^−1^)	0.21	0.21	0.23	0.20	0.15
*P*_TAG, ground_ (*t*)^a^ (g m^−2^ day^−1^)	2.46	1.91	2.06	2.26	0.97
*Y*_TAG, ph, max_ (*t*) (g mol^−1^ photons)	0.07 (3)	0.06 (3)	0.09 (6)	0.09 (5)	0.06 (4)
*E*_ground_ (*t*) (mol photons m^−2^ day^−1^)	39	36	23	24	18

^a^At maximum time-averaged TAG yield per mole photons

#### Outdoor semi-continuous cultivations

**Fig. 4:**
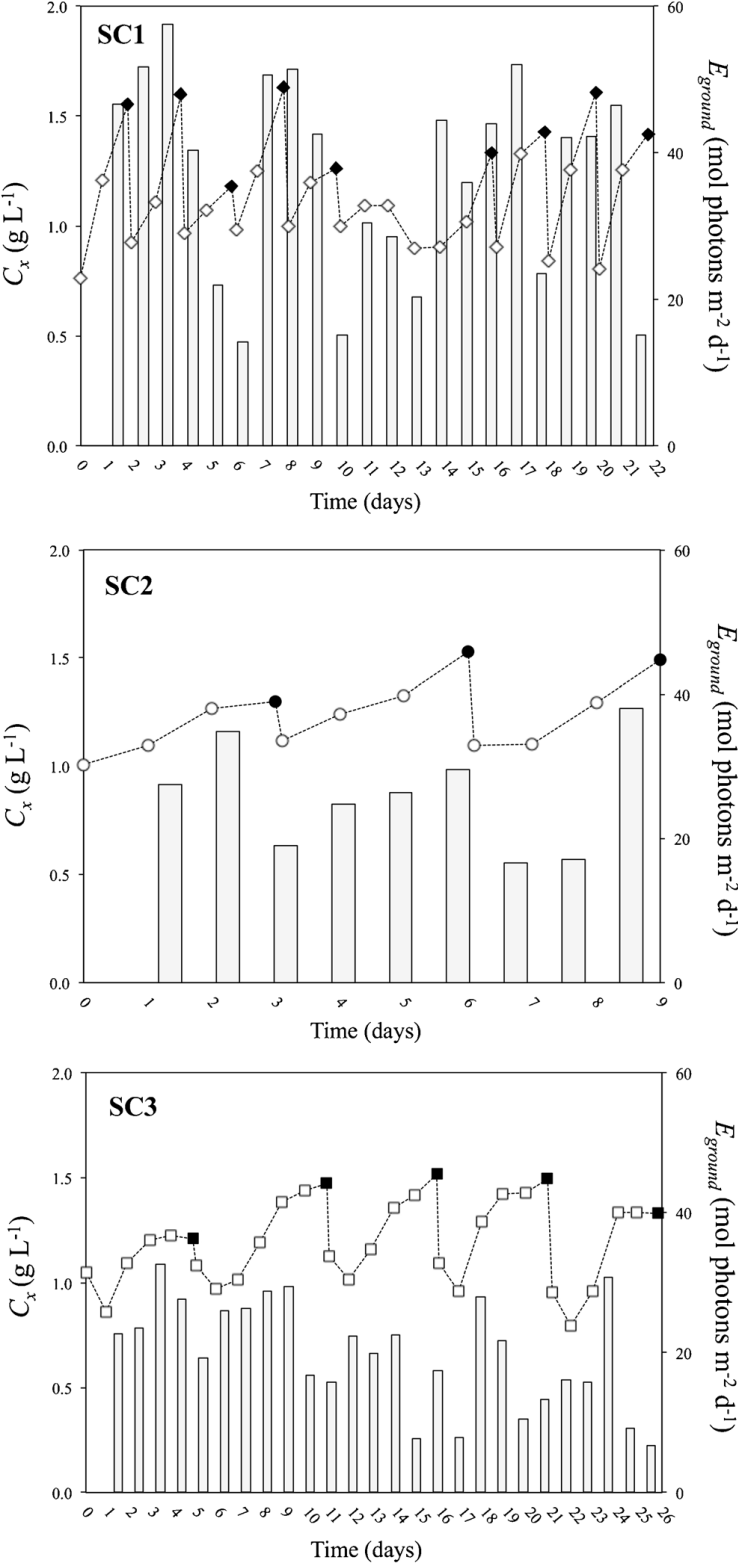
Time-evolution of biomass concentration (*C*_*x*_; *symbols*) and daily irradiance on ground area (*E*_ground_; *bars*) for the outdoor semi-continuous runs. SC1, SC2 and SC3 were harvested every 1, 2 and 3 days after nitrogen-depletion, respectively. *Black symbols* correspond to days at which a harvest was applied. Lines are drawn only for illustrative purposes

In contrast with the lab-scale semi-continuous experiments, the biomass concentration at harvest greatly varied for the outdoor semi-continuous runs (1.18–1.63, 1.30–1.53 and 1.21–1.52 g L^−1^) for SC1, SC2 and SC3, respectively (Fig. 4; symbols) because of varying light conditions (Fig. 4; bars). Therefore, constant cycle repetitions were not achieved. In SC1 and SC2, nitrogen (N) was generally consumed within 24 h from addition. However, at lower total irradiance, i.e. SC3, N was depleted from the medium only after 2 or 3 days from addition. This resulted in longer re-growth phases, which strongly reduced the time-averaged biomass and TAG productivities and yields per mole photons (Table 5). Average TAG contents at harvest were low (10–13 % *w*/*w*) and similar among the different semi-continuous runs (Table 5). Throughout a single semi-continuous run, TAG yield per mole photons (*Y*_TAG, ph, cycle_) greatly differed over the cycles (Supplementary material 2).

**Table 5:** Time-averaged ground areal biomass (*P*_*x*, ground_ (*t*)) and TAG (*P*_TAG, ground_ (*t*)) productivities, time-averaged biomass (*Y*_*x*, ph_ (*t*)) and TAG (*Y*_TAG, ph, max_ (*t*)) yields per mole photons, TAG content (*f*_TAG_) and time-averaged light intensity (*E*_ground_ (*t*)) for the outdoor semi-continuous (SC) runs. SC1, SC2 and SC3 were harvested every 1, 2 and 3 days after nitrogen-depletion, respectively. For *f*_TAG_, mean and standard deviation are reported (SC1, *n* = 9; SC2, *n* = 3; SC3, *n* = 5)

Outdoor semi-continuous runs			
	SC1	SC2	SC3
*P*_*x*, ground_ (*t*) (g m^−2^ day^−1^)	8.13	4.77	2.66
*Y*_*x*, ph_ (*t*) (g mol^−1^ photons)	0.22	0.18	0.13
*f*_TAG_ (g g^−1^)	0.10 ± 0.05	0.13 ± 0.02	0.13 ± 0.01
*P*_TAG, ground_ (*t*) (g m^−2^ day^−1^)	0.85	0.59	0.35
*Y*_TAG, ph_ (*t*) (g mol^−1^ photons)	0.022	0.023	0.018
*E*_ground_ (*t*) (mol photons m^−2^ day^−1^)	37	26	20

## Discussion

### Batch vs. semi-continuous TAG production at lab-scale

The TAG productivity obtained for the constant semi-continuous cycle repetitions (#1–#3) is compared with the maximum time-averaged batch TAG productivity (i.e. the productivity at the optimal harvest time for the batch; *P*_TAG, max_ (*t*)). Noteworthy, the TAG productivity obtained with shortest semi-continuous cycle (SC1; 0.21 g L^−1^ day^−1^) was similar to the maximum TAG productivity of the batch process (i.e. optimal harvest time for the batch; 0.23 g L^−1^ day^−1^) (Table 3). Although, SC1 resulted in a lower TAG content (0.22 g g^−1^) compared to the batch (0.35 g g^−1^), much higher biomass productivity was obtained with SC1. About 0.73 g L^−1^ day^−1^ of non-TAG-biomass was produced in SC1, whereas 0.43 g L^−1^ day^−1^ was produced in the batch. Several cellular components can contribute to the non-TAG-fraction of the biomass, such as non-acyl lipids, glyco- and phospholipids, sugars and proteins (Wang and Wang 2012; Bondioli et al. 2012). For instance, with the calculated intracellular nitrogen content (Supplementary material 1), it is possible to estimate the mass fraction and productivity of proteins (Breuer et al. 2012) that, besides TAGs, represent one of the major biomass constituents with a high economic value (Wijffels et al. 2010). The batch cultivations resulted in an estimated protein content of 0.21 g g^−1^ with a productivity of 0.14 g L^−1^ day^−1^. SC1 yielded similar protein contents (0.24 g g^−1^) but higher productivities (0.22 g L^−1^ day^−1^). Therefore, if only the TAG fraction of the biomass is used, the lower TAG contents obtained with semi-continuous processes will likely result in higher costs for downstream operations (harvesting, dehydration, extraction) (Molina-Grima et al. 2003). Semi-continuous TAG production may become cost effective if a biorefinery approach is pursued and the whole biomass is valorized (Wijffels et al. 2010). For this, mild cell disruption techniques (e.g. pulsed electric field) and separation technologies (e.g. ionic liquids), which are able to both separate hydrophobic and hydrophilic compounds, should be adopted (Vanthoor-Koopmans et al. 2013).

When comparing the TAG yields per mole photons obtained in this study with those reported in literature, higher values were found for *Chlorella* and *Scenedesmus* cultivations in flat panel reactors to which lower (175–500 μmol photons m^−2^ s^−1^) and continuous light intensities were supplied (Han et al. 2013; Breuer et al. 2014; Mulders et al. 2014). Besides that the TAG yield per mole photons of different species differs substantially (Griffiths and Harrison 2009; Breuer et al. 2012; Benvenuti et al. 2014), the lower yields found in our study under day/night cycles may be explained by the very high incident light intensities experienced during the central hours of the day (up to 1500 μmol photons m^−2^ s^−1^). It is indeed known that very high light intensities result in substantial yield losses, whereas lower incident light intensities are beneficial for TAG production (Breuer et al. 2013b). Additionally, during the night, energy storage metabolites are likely be respired to satisfy the maintenance energy demand (Torzillo et al. 1991; Fábregas et al. 2002), thus further decreasing the TAG yield per mole photons.

### Batch vs. semi-continuous TAG production in outdoor photobioreactors

**Fig. 5:**
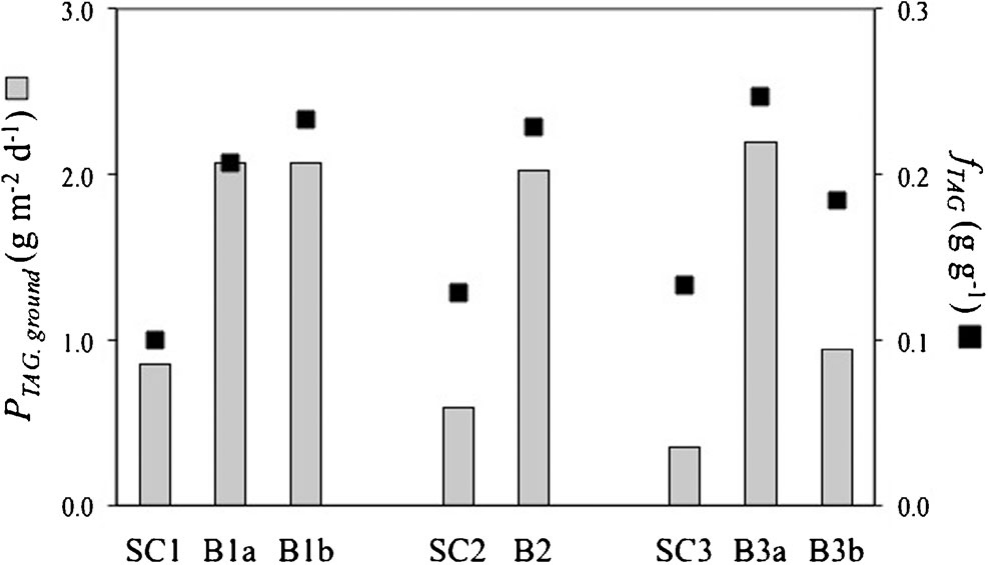
TAG content (*f*_TAG_) and time-averaged ground areal TAG productivity (*P*_TAG, ground_) of the outdoor batch (B1*a*, B1*b*, B2, B3*a*, B3*b*) and semi-continuous (SC1, SC2, SC3) cultivations. For process comparison, *P*_TAG, ground_ was calculated over the same period (22, 9 or 26 days) for both operational strategies

As constant cycle repetitions were not achieved with the semi-continuous runs (Fig. 4), outdoor batch and semi-continuous TAG production processes are compared on the TAG productivity calculated over the same period (Fig. 5). The period considered for comparing the batch with the corresponding semi-continuous process is equivalent to the duration of the semi-continuous cultivation. It is assumed that, during that period, the batch culture is harvested when the time-averaged TAG yield per mole photons is maximal (i.e. optimal harvest time) and started again (section 2.4.1). The batch productivities were re-calculated over 22 days (for B1*a* and B1*b*), 9 days (for B2) and 26 days (for B3*a*, B3*b*) and then compared to the time-averaged TAG productivities of the semi-continuous process. By harvesting the batch cultures when the time-averaged TAG productivity is maximal, much higher TAG productivities (and contents) can be achieved with batch than with semi-continuous cultivations (Fig. 5). In the latter ones, likely, too much nitrogen was present in the system for the given irradiance and cycle duration.

The highest TAG yield per mole photons achieved with our semi-continuous cultivations (*Y*_TAG, ph, cycle_ of 0.04 g mol^−1^ photons corresponding to a *P*_TAG, cycle_ of 2.2 g m^−2^ day^−1^ for SC1) was about 1.5-fold lower than the highest *Y*_TAG, ph, cycle_ (0.06 g mol^−1^ photons corresponding to a *P*_TAG, cycle_ of 7.7 g m^−2^ day^−1^) reported by Bondioli et al. (2012) for a semi-continuous cultivation (44 % daily harvest) of *Nannochloropsis* sp. This discrepancy mainly relies on the higher degree of stress applied to cells in the study of Bondioli et al. (2012) compared to our semi-continuous cultivations. Firstly, in their study, no nitrogen (N) was re-supplied after harvest. Secondly, the culture likely experienced higher light availabilities. Because of the higher total irradiance and the reactor used by Bondioli et al. (2012) (i.e. single flat panel not subjected to mutual shadowing from other panels, as was the case for the tubes used in our study), their culture received a higher amount of light. Thus, the combination of lower nitrogen in the system and higher light availabilities resulted in the higher *Y*_TAG, ph, cycle_ reported by Bondioli et al. (2012).

Noteworthy, the maximum time-averaged TAG yields per mole photons (Table 4) obtained in our outdoor batch cultivations are comparable with the ones found by Quinn et al. (2012) with the same species cultivated year-round in outdoor flat panel reactors in Colorado, USA.

### Outlook on future research

To certainly assess whether semi-continuous TAG production represents an effective alternative to batch processes, several aspects should be further investigated.

Outdoors, focus should be put on the applied stress pressure, and both nitrogen (N) supply and harvest frequency should be adjusted based on the expected total irradiance. Accurate production models could contribute in identifying optimal “nitrogen-to-light ratios”. These models should be developed based on dedicated sets of lab-scale experiments, where the dependency of both N-supply and harvest frequency from irradiance is investigated under well-defined light regimes. Subsequently, the models should be validated outdoors under varying light conditions. Regardless of the operational strategy, daily measurable parameters such as the irradiance, the biomass concentration (e.g. turbidity) and the cellular TAG content (e.g. Nile Red fluorescence (Chen et al. 2009) or FTIR spectroscopy (Miglio et al. 2013; Mayers et al. 2013)) should be used to implement the optimal operational settings in such a way that harvest is always appropriately timed for any given N-supply and any given irradiance.

Additionally, to operate an optimized semi-continuous process, a full understanding of cell recovery mechanisms upon N-replenishment is required as these may greatly affect the productivity of the entire process. Only few research papers have been published on this topic (Siaut et al. 2011; Fernandes et al. 2013; Mulders et al. 2015). In these studies, cells were replenished with an excess of nitrogen after a long N-starvation period (>7–15 days). It was found that the TAGs, which were accumulated during the N-starvation period, were entirely respired within 2 days from N-replenishment to fuel the recovery process. However, the extent of TAG degradation and its rate depend on several factors such as species-specific photosynthetic responses to N-starvation and recovery, amount of re-supplied nitrogen, harvest frequency and harvest volume. Therefore, the dependency of recovery mechanisms on these factors has to be fully understood before an optimal semi-continuous process can be designed.

Finally, as also speculated by Mulders et al. (2015), higher outdoor semi-continuous TAG productivities could possibly be achieved by resupplying the nitrogen around sunset. Culture recovery would then occur at night (Siaut et al. 2011; Přibyl et al. 2013), thereby enhancing TAG production during the light period.

### Conclusions

The lab-scale experiments demonstrated that semi-continuous strategies could achieve similar TAG productivities compared to batch processes. Additionally, it was shown that semi-continuous cultivations can potentially make TAG production cost effective by valorising also non-TAG-compounds provided that biorefinery of the whole biomass is pursued. Contrarily, further optimization of outdoor semi-continuous strategies is necessary as these were always outcompeted by the batch process. In particular, attention should be given to the chosen semi-continuous operational settings (e.g. nitrogen supply and harvest frequency) as these, together with the total irradiance, determine the applied stress pressure and thus, the productivity of the process.
